# Neuropeptide modulation of lymphatic smooth muscle tone in the canine forelimb

**DOI:** 10.1155/S096293519200036X

**Published:** 1992

**Authors:** David E. Dobbins

**Affiliations:** Department of Physiology Uniformed Services University of the Health Sciences 4301 Jones Bridge Road Bethesda MD 20814-4799 USA

## Abstract

Neurokinin A and B are putative inflammatory mediators. We assessed their ability to alter prenodal lymphatic resistance. Intralymphatic neurokinin A (3.0 × 10^−6^, 3.0 × 10^−5^ and 3.0 × 10^−4^ mol l^−1^) significantly constricted lymphatics at the two highest doses. Preliminary experiments suggested that neurokinin B might dilate lymphatics. To test this, lymphatic pressure was increased by norepinephrine (3.1 × 10^−6^ mol l^−1^). Neurokinin B (2.7 × 10^−4^ mol l^−1^) was then infused intralymphatically during norepinephrine infusion. Norepinephrine increased perfusion pressure from 5.6 ± 0.6 mmHg to 12.1 ± 1.4 mmHg. Subsequent infusion of neurokinin B significantly decreased lymphatic perfusion pressure from 11.9 ± 1.3 mmHg to 9.9 ± 1.1 mmHg. These data indicate that neurokinin A and B can alter lymphatic resistance and are consistent with the hypothesis that lymph vessel function may be subject to modulation by neurokinins.


Mediators of Inflammation, 1, 241–246 (1992)

### Neuropeptide modulation of lymphatic smooth muscle tone in the canine forelimb

David E. Dobbins

Department of Physiology, Uniformed Services University of the Health Sciences, 4301 Jones Bridge Road, Bethesda, MD 20814-4799, USA

CA Corresponding Author

**NEUROKININ A and B are putative inflammatory mediators. We assessed their ability to alter prenodal lymphatic resistance. Intralymphatic neurokinin A ($3.0 \times 10^{-6}$, $3.0 \times 10^{-5}$ and $3.0 \times 10^{-4}$ mol $l^{-1}$) significantly constricted lymphatics at the two highest doses. Preliminary experiments suggested that neurokinin B might dilate lymphatics. To test this, lymphatic pressure was increased by norepinephrine ($3.1 \times 10^{-6}$ mol $l^{-1}$). Neurokinin B ($2.7 \times 10^{-4}$ mol $l^{-1}$) was then infused intralymphatically during norepinephrine infusion. Norepinephrine increased perfusion pressure from $5.6 \pm 0.6$ mmHg to $12.1 \pm 1.4$ mmHg. Subsequent infusion of neurokinin B significantly decreased lymphatic perfusion pressure from $11.9 \pm 1.3$ mmHg to $9.9 \pm 1.1$ mmHg. These data indicate that neurokinin A and B can alter lymphatic resistance and are consistent with the hypothesis that lymph vessel function may be subject to modulation by neurokinins.**

**Key words:** Canine forelimb, Lymphatic constriction, Lymphatic resistance, Lymphatic smooth muscle, Lymph vessel function, Neurokinins, Tachykinins.

### Introduction

Neurokinin A (also known as substance K) and neurokinin B (also known as neuromedin K) are both ten amino acid peptides of the tachykinin family which were originally isolated from mammalian spinal cord by Kimura *et al.*[1] in 1983. The distribution of the neurokinins in both the central and peripheral nervous systems has been reported to be similar to that of substance P, the prototype of the tachykinin family.[2] The concentration of these peptides in perivascular nerve terminals impinging upon the resistance vessels of the peripheral circulation has led to the suggestion that they play a role in the modulation of the circulation.[3,4] Additionally, evidence is accumulating which suggests that alterations in tachykinin levels may play a role in a multitude of pathophysiological conditions.[3,5–10]

We have previously shown that the intra-arterial infusion of nanogram quantities of neurokinin A and B results in potent vasodilation in the skin and skeletal muscle circulations of the canine forelimb.[11] Although the vascular actions of these neuropeptides have begun to be delineated, no work has yet been published concerning the possible impact of these agents on lymphatic vessel function via alterations in lymphatic smooth muscle tone. Previous reports have revealed that the central lacteal lymphatics of canine ileal and duodenal villi[12,13] and the lymphatic capillaries in rat liver[14] are innervated with nerves which contain tachykinins. These findings indicate that these vessels might be subject to control, at least in part, by tachykinins.

A wide array of endogenous vasoactive agents including catecholamines,[15] autacoids,[16] inflammatory mediators,[16] prostanoids[17] and endothelin[18] are capable of increasing lymphatic resistance when administered either intralymphatically or intra-arterially. In the current study we assessed the ability of intralymphatically infused neurokinin A and B to alter lymphatic smooth muscle tone in perfused prenodal lymphatic vessels.

### Materials and Methods

Adult mongrel dogs of either sex were anaesthetized with sodium pentobarbital (35 mg $kg^{-1}$ i.v. and supplemented as needed), intubated and ventilated with room air. Small incisions were made in the skin of the right forelimb and the brachial artery. A small side branch of the brachial artery and a skin small vein and artery in the paw were isolated. The forelimb was perfused at constant arterial inflow via the brachial artery with blood obtained from a cannulated femoral artery. Forelimb perfusion pressure was measured from the side branch of the brachial artery, and skin small vein and artery pressures were measured on the dorsal and ventral surfaces of the paw respectively. Systemic arterial pressure was measured by inserting a catheter into the brachial artery and advancing it into the aorta. A catheter was inserted into the left external jugular vein and advanced to

© 1992 Rapid Communications of Oxford Ltd 

the level of the right atrium for measurement of central venous pressure.

A lymph vessel on the dorsal surface of the paw was cannulated in the direction of normal lymph flow. The lymph vessel was perfused at constant flow at a volume flow rate of 0.034 ml $min^{-1}$ with a perfusate which was the supernatant of a 1:1 mixture of autologous arterial blood and heparinized Krebs solution. Lymphatic perfusion pressure was measured from the perfusion system at a point upstream to the point of cannulation of the prenodal lymph vessel. A three-way stopcock, which had been modified such that all three ports were confluent, allowed for measurement of lymphatic perfusion pressure and for the lymphatic to be perfused with either control perfusate or perfusate containing tachykinins.

The protocol was as follows: in all experiments, the lymph vessel was perfused with control perfusate for a minimum of 15 min to ensure that all measured pressures had reached steady state values. In the neurokinin A experiments, the lymph vessel was then perfused with a solution containing neurokinin A at $3.0 \times 10^{-6}$, $3.0 \times 10^{-5}$ or $3.0 \times 10^{-4}$ mol $l^{-1}$ for a minimum of 15 min or until the peak response in lymphatic perfusion pressure had been obtained. The lymphatic was then perfused with control perfusate until the lymphatic perfusion pressure returned to control values. Early experiments revealed that neurokinin A shares, with histamine and angiotensin II, the characteristic of altering lymphatic perfusion pressure upon its initial infusion but thereafter renders the lymph vessel insensitive to infusion of the same agent even at concentrations several orders of magnitude higher. Therefore, the three point dose-response relationship to neurokinin A had to be generated in three separate ($n = 7$) series of animals to ascertain the actual potency of this agent in altering prenodal lymphatic resistance.

Preliminary experiments with the intralymphatic infusion of neurokinin B suggested that this peptide would likely dilate lymphatic vessels. Since the control pressure in these vessels as perfused in this study is relatively low (4–5 mmHg on average) it appeared best to first increase the lymphatic perfusion pressure with the intra-arterial infusion of a known lymphatic constrictor agent and then infuse neurokinin B intralymphatically in the face of the steady but elevated lymphatic perfusion pressure. To this end, in the neurokinin B experiments, norepinephrine was infused intra-arterially at a concentration of $3.1 \times 10^{-6}$ mol $l^{-1}$. When the lymphatic perfusion pressure had reached a steady elevated value, neurokinin B was infused intralymphatically at a concentration of $2.7 \times 10^{-4}$ mol $l^{-1}$ during the continued intra-arterial infusion of norepinephrine. After the peak response to neurokinin B had been obtained, the lymphatic was again perfused with control perfusate while the intra-arterial infusion of norepinephrine continued. This manoeuvre was performed to ensure that the decrease in lymphatic perfusion pressure seen during neurokinin B infusion was indeed caused by neurokinin B and not merely a dissipation of the effects of norepinephrine. When lymphatic perfusion pressure had again reached a steady state, the intra-arterial infusion of norepinephrine was discontinued and lymphatic perfusion pressure was allowed to return to control values.

The effects of a single dose of substance P, the prototype of the tachykinin peptide family, was also tested for its ability to alter lymphatic resistance. Following acquisition of control values, substance P at a concentration of $2.4 \times 10^{-4}$ mol $l^{-1}$ was infused intralymphatically for a minimum of 15 min or until the peak response was obtained. Following the conclusion of the substance P infusion, the lymphatic vessel was again perfused with control perfusate until all measured pressures returned to control values.

Norepinephrine, neurokinin A, neurokinin B and substance P were made up fresh daily in normal saline in a stock solution of 1.5 mg $ml^{-1}$. Final dilutions of norepinephrine were made in normal saline and it was infused into the arterial blood supply to the forelimb with a needle tipped catheter. Final dilutions of the tachykinins were made by adding the appropriate amount of the lymphatic perfusate. All data were analysed using Student's $t$ test as modified for paired replicates. Pressures obtained immediately prior to an experimental manoeuvre were compared with those achieved during the peak of the response seen during the manoeuvre.

### Results

The infusion of neurokinin A at the lowest infusion rate did not significantly alter lymphatic perfusion pressure (Figure 1) from its control value of $5.7 \pm 0.6$ mmHg. However, infusion of the middle dose of neurokinin A significantly increased lymphatic pressure from a control value of $6.7 \pm 0.8$ mmHg to a peak pressure of $8.0 \pm 0.8$ mmHg. The highest dose of neurokinin A resulted in a doubling of lymphatic resistance, increasing lymphatic perfusion pressure from a control value of $6.3 \pm 0.9$ mmHg to a peak value of $12.3 \pm 1.7$ mmHg. It should be remembered that these data were generated in three separate groups of animals and thus variations in the control lymphatic perfusion pressure do not reflect an instability within the preparations with time but rather indicate minor differences in control

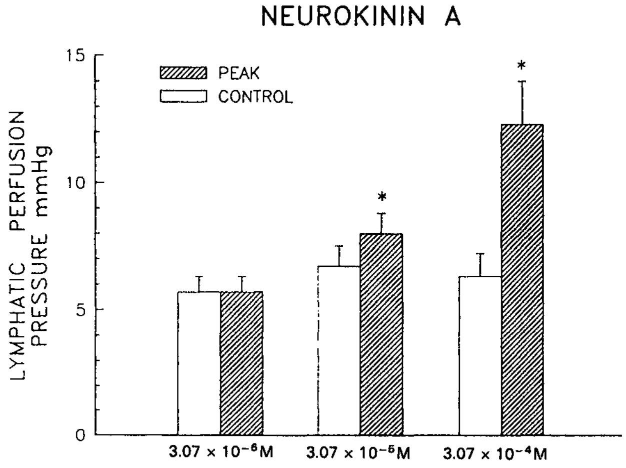

FIG. 1. Effects of the intralymphatic infusion of neurokinin A on lymphatic perfusion pressure. $n = 7$ in three separate groups of animals. * = $p \leq 0.05$, Student's paired $t$.

lymphatic resistances between different groups of animals. The intralymphatic infusion of neurokinin A did not significantly alter either mean systemic arterial, central venous or forelimb perfusion, small artery or vein pressures (Table 1).

In the neurokinin B experiments, the intra-arterial infusion of norepinephrine resulted in a significant increase in lymphatic perfusion pressure from a control value of 5.6 ± 0.6 mmHg to a peak pressure of 12.1 ± 1.4 mmHg (Figure 2). Systemic pressure, forelimb perfusion, skin small artery and vein pressures were all significantly increased (Table 2). The intralymphatic infusion of neurokinin B during the continued infusion of norepinephrine resulted in a significant decrease in lymphatic perfusion pressure from its pre-neurokinin level of 11.9 ± 1.3 mmHg to a nadir of 9.9 ± 1.1 mmHg (Figure 2). Intralymphatic infusion of neurokinin B did not affect significantly any of the measured vascular pressures (Table 2). When the lymphatic was again perfused with control perfusate during the continued infusion of norepinephrine, lymphatic perfusion pressure returned to its pre-neurokinin levels. This indicated that the decrease observed during infusion of neurokinin B was not simply a waning of the effects of norepinephrine on the lymphatic. When the intra-arterial infusion of norepinephrine was terminated, lymphatic perfusion pressure returned rapidly to its pre-norepinephrine level.

The intralymphatic infusion of substance P at $2.4 \times 10^{-4}$ mol $l^{-1}$ ($n = 6$) did not significantly change lymphatic perfusion pressure from its control value of 4 ± 0.4 mmHg and did not significantly alter any of the measured vascular pressures.

### Discussion

The localization of neurokinins in perivascular nerve terminals innervating the peripheral circulation has led to the suggestion that these endogenous peptides may modulate the peripheral circulation under either normal or pathophysiological conditions. However, a role for these potent vasoactive agents in health or disease has not yet been established. The increase in tachykinin levels in the synovial fluid of patients with osteoarthritis and rheumatoid arthritis,[19] and the known role of substance P, the prototype of the tachykinin family, in neurogenic inflammation[20-22] has led to the suggestion that these agents might alter transvascular fluid and macromolecular flux. Gamse and Saria[7] have reported that neurokinin A and B produce plasma protein extravasation in rat abdominal skin and Fuller *et al.*[23] noted that the neurokinins produce a weal and flare when injected into human skin. However, preliminary experiments conducted in our laboratory indicated that the neurokinins do not increase skin lymph flow or its protein concentration in the canine forelimb as would be expected with agents which increase microvascular permeability. These data are consistent with previously published experiments[24] which indicated

**Table 1.** Effect of intralymphatic neurokinin A infusion on vascular pressures. $n = 7$ in each of three separate groups

| | $3.0 \times 10^{-6}$ | | $3.0 \times 10^{-5}$ | | $3.0 \times 10^{-4}$ | |
|---|---|---|---|---|---|---|
| | C | P | C | P | C | P |
| Sys | 128 ± 4.6 | 128 ± 4.1 | 128 ± 2.2 | 127 ± 2.3 | 130 ± 2.5 | 129 ± 2.5 |
| Perf | 109 ± 5.6 | 112 ± 6.2 | 110 ± 3.3 | 113 ± 3.9 | 117 ± 6.0 | 116 ± 4.9 |
| Ssa | 80 ± 3.2 | 81 ± 3.2 | 88 ± 3.9 | 91 ± 5.1 | 94 ± 3.3 | 94 ± 2.8 |
| Ssv | 9 ± 0.6 | 9 ± 0.6 | 10 ± 0.3 | 10 ± 0.3 | 9 ± 0.6 | 9 ± 0.6 |
| Cv | 2 ± 0.1 | 2 ± 0.1 | 3 ± 0.5 | 3 ± 0.5 | 4 ± 0.5 | 4 ± 0.4 |

Sys = mean systemic, Perf = forelimb perfusion, Ssa = skin small artery, Ssv = skin small vein, Cv = central venous. Pressures are means ± SEM expressed in mmHg. * = $p \leq 0.05$ Student's paired $t$. C = control, P = peak pressure. Concentration of neurokinin A is in mol $l^{-1}$.

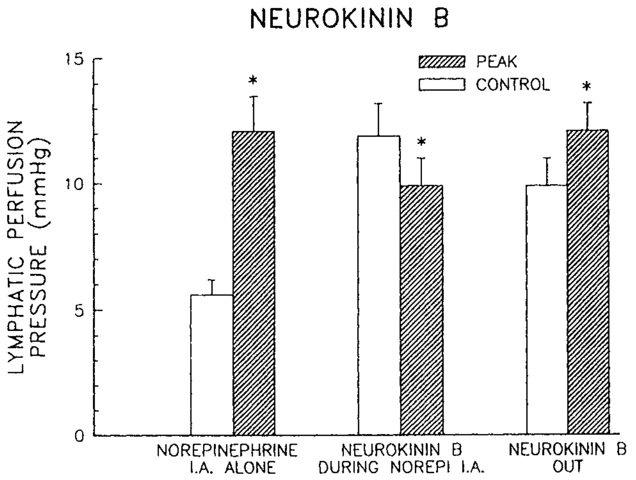

FIG. 2. Effects of the intra-arterial infusion of norepinephrine at $3.1 \times 10^{-6}$ mol $l^{-1}$ and intralymphatic infusion of neurokinin B at $2.7 \times 10^{-4}$ mol $l^{-1}$ during continued norepinephrine infusion on lymphatic perfusion pressure. $n = 6$, * = $p \leq 0.05$, Student's paired *t*.

that substance P likewise fails to increase lymph flow and its protein concentration in the canine forelimb. However, vasoactive agents can also alter fluid transport by altering the lymphatic vessel's ability to transport fluid. In light of the fact that previous experiments have revealed that some lymphatic vessels are innervated with tachykinin containing nerves,[12–14] this study was undertaken to ascertain if neurokinins could influence fluid dynamics through alterations in lymphatic smooth muscle tone.

The experiments in this study clearly indicate that both neurokinin A and neurokinin B are capable of altering lymphatic resistance. The intralymphatic infusion of neurokinin A results in significant increases in lymphatic resistance with a threshold dose between $3.0 \times 10^{-6}$ and $3.0 \times 10^{-5}$ mol $l^{-1}$. At the highest infusion rate utilized, neurokinin infusion results in a doubling of lymphatic resistance. We have previously reported that a wide array of vasoactive agents are capable of constricting prenodal lymphatic vessels in the canine forelimb.[15–18] The threshold concentrations at which significant lymphatic constriction is manifest varies widely among vasoactive agents, from the extremely potent lymphatic constrictor endothelin (threshold concentration between $10^{-10}$ and $10^{-9}$ mol $l^{-1}$) to markedly less potent agents such as prostaglandin $E_1$ (threshold concentration between $10^{-4}$ and $10^{-3}$ mol $l^{-1}$). The current study indicates that the threshold concentration of neurokinin A required to obtain significant lymphatic constriction ($10^{-6}$ to $10^{-5}$ mol $l^{-1}$) lies in the middle range of tested agents and is comparable to that seen with bradykinin, dopamine and acetylcholine. However, precise conclusions as to the potency of neurokinin A as a lymphatic constrictor are difficult to make. Since neurokinins are located in nerve endings, the lymphatic perfusate concentrations of these compounds which are reported in this study to be effective in altering lymphatic smooth muscle tone may not accurately reflect their true effective concentrations at their effector site on the lymphatic smooth muscle. The effective concentrations at the point of release on the lymphatic smooth muscle cell may well be substantially lower than effective circulating concentrations.

The current study also indicates that intralymphatic infusion of neurokinin B is capable of producing a significant decrease in lymphatic resistance. This is best seen, in light of the control lymphatic perfusion pressures involved, in pre-constricted lymph vessels. In this respect, neurokinin B is similar in action to the $\beta_2$-receptor agonist terbutaline[25] and to adenosine which also significantly relax pre-constricted lymphatics. The concentration of neurokinin B which is needed to produce significant relaxation of pre-constricted lymphatic vessels is similar to that required for both adenosine and terbutaline.

Preliminary studies with substance P in this experimental model had suggested that it did not significantly alter lymphatic perfusion pressure. In this study, to clarify that point, we assessed the actions of a single dose of substance P given intralymphatically. Indeed, as suspected, substance P did not significantly alter lymphatic perfusion pressure. Therefore, it appears that while neurokinin A and neurokinin B administered intralymphatically in adequate doses can alter lymphatic resistance, substance P in comparable doses does not. Since substance P, neurokinin A and neurokinin B are known to be the preferred ligands for the NK-1, NK-2 and NK-3 tachykinin receptors respectively, it could be speculated that the prenodal lymph vessels in the canine forelimb contain NK-2 and NK-3 receptors but not NK-1 receptors. However, this cannot be determined by the results of the current study and will require

**Table 2.** Effects of intra-arterial norepinephrine and intralymphatic neurokinin B on vascular pressure, $n = 6$

| | Norepinephrine | | Neurokinin B | |
|---|---|---|---|---|
| | C | P | C | P |
| Sys | 124 ± 6.8 | 131* ± 6.7 | 127 ± 6.4 | 127 ± 6.4 |
| Perf | 117 ± 8.0 | 225* ± 10.0 | 206 ± 7.3 | 205 ± 7.5 |
| Ssa | 78 ± 9.8 | 179* ± 12.9 | 157 ± 13.2 | 157 ± 13.2 |
| Ssv | 11 ± 0.7 | 19* ± 3.3 | 16 ± 1.5 | 16 ± 1.5 |
| Cv | 4 ± 0.01 | 4 ± 0.01 | 4 ± 0.01 | 4 ± 0.01 |

Sys = mean systemic, Perf = forelimb perfusion, Ssa = skin small artery, Ssv = skin small vein, Cv = central venous. All pressures are means ± SEM expressed in mmHg. * = $p \leq 0.05$ Student's paired *t*. C = control, P = peak pressure.

additional experimentation utilizing specific neurokinin-receptor agonists.

As stated previously, a precise physiological role for the neurokinins has not yet been established firmly but several lines of evidence have implicated these peptides in control of the peripheral circulation. Sann *et al.*[4] noted that the plantar capillary blood flow in rats was significantly less in a leg whose sciatic nerve had been treated with capsaicin (an agent known to deplete nerves of tachykinins) than in the contralateral untreated leg. They thus concluded that capsaicin sensitive peptidergic afferents may contribute to regional blood flow control in the skin. Neurokinins may also play a role in the nonadrenergic noncholinergic excitatory control of the airways. Thompson *et al.*[26] reported that tachykinin depletion significantly modifies the nonadrenergic, noncholinergic excitatory responses in guinea-pig trachea.

A role for the tachykinins in pathophysiological states has also been proposed. Barnes[5] has suggested that these neuropeptides may be involved in neurogenic inflammatory reactions in asthma. Uchida *et al.*[27] noting that neurokinin A is released by chemical irritants such as cigarette smoke suggested that neurokinin A and other tachykinins may mediate the subendothelial oedema caused by cigarette smoke. Lundin *et al.*[28] reported that the severity of carcinoid heart disease was correlated to the degree to which plasma levels of serotonin and tachykinins were elevated. It has been suggested that tachykinin-mediated stimulation of fibroblasts may increase fibrosis of the valves in carcinoid heart disease. Krause *et al.*[8] reported that the circulating levels of substance P more than triple in haemorrhagic shock. Since neurokinin A and neurokinin B appear to be co-localized with substance P and are released by common stimuli such as capsaicin, it is likely that neurokinin levels would likewise be elevated in shock.

The results of previously published work clearly indicate that neurokinin A and neurokinin B have potent cardiovascular effects. Their potent vasodilatory actions make them candidate modulators of the peripheral circulation in either health or numerous disease states. Additionally, the current study suggests that they may also play a role in control of lymphatic vessel function via alterations in lymphatic smooth muscle tone. By way of their actions to influence the ability of the lymph vessels to transport fluid, these agents could impact transvascular fluid flux and oedema formation. In addition, their potent vasodilatory effects could act to supplement the increase in transvascular fluid and macromolecular flux produced by inflammatory mediators through an increase in blood flow and perfused capillary surface area. However, additional studies will be required before a firm role for the neurokinins can be established. Measurement of circulating levels of neurokinins under various pathophysiological conditions is needed to determine under what conditions these agents are released and to determine what circulating concentrations are obtained. Also, a continued refinement in the development of specific neurokinin receptor agonists, antagonists and specific releasing agents is required to firmly establish the role of these neuropeptides in modulation of the peripheral circulation under a myriad of conditions.

### References

1. Kimura S, Okada M, Sugita Y, Kanazawa I, Munekata E. Novel neuropeptides, neurokinin $\alpha$ and $\beta$ isolated from porcine spinal cord. *Proc Japan Acad* 1983; **59Ser. B**: 101–104.
2. Kangawa K, Minamino N, Fukuda A, Matsuo H. Neuromedin K: A novel mammalian tachykinin identified in porcine spinal cord. *Biochem Biophys Res Commun* 1983; **114(2)**: 533–540.
3. Maggio JE. Tachykinins. *Ann Rev Med* 1988; **11**: 13–28.
4. Sann H, Pinter E, Szolcsanyi J, Pierau Fr-K. Peptidergic afferents might contribute to the regulation of skin blood flow. *Agents & Actions* 1988; **23**: 14–15.
5. Barnes PJ. Neuropeptides in human airways: Function and clinical implications. *Am Rev Respir Dis* 1987; **136**: S77–S83.
6. Forman JV. Neuropeptides and the pathogenesis of allergy. *Allergy* 1987; **42**: 1–11.
7. Gamse R, Saria A. Potentiation of tachykinin-induced plasma protein extravasation by calcitonin gene-related peptide. *Eur J Pharmacol* 1985; **114**: 61–66.
8. Krause W, Rathsack R, Oehme P. Substance P (SP) and hemorrhagic shock. *Prog Brain Res* 1986; **6**: 106.
9. Lewis DA, Bloom FE. Clinical perspectives on neuropeptides. *Ann Rev Med* 1987; **38**: 143–148.
10. Strubble RG, Powers RE, Casanova MF, Kitt CA, Brown EC, Price DL. Neuropeptidergic systems in plaques of Alzheimer's disease. *J Neuropathol Exp Neurol* 1987; **46(5)**: 567–584.
11. Dobbins DE, Buehn MJ, Dabney JM. Neurokinin A and B: Potent vasodilators in the canine forelimb. *Microcirc Endoth Lymphatics* 1990; **6**: 253–266.
12. Ichikawa S, Kasahara D, Iwanaga T, Uchino S, Fujita T. Peptidergic nerve terminals associated with the central lacteal lymphatics in ileal villi of dogs. *Arch Histol Cytol* 1991; **54(3)**: 311–320.
13. Ichikawa S, Shiozawa M, Iwanaga T, Uchino S. Immunohistochemical demonstration of peptidergic nerve fibers associated with the central lymphatics of the duodenal villi of dogs. *Arch Histol Cytol* 1991; **54(2)**: 241–248.
14. Ito Y, Magari S, Sakanaka M. Immunoelectron-microscopic localization of peptidergic nerve fibers around lymphatic capillaries in the rat liver. *Arch Histol Cytol* 1990; **53(Suppl)**: 199–208.
15. Dabney JM, Buehn MJ, Dobbins DE. Constriction of lymphatics by catecholamines, carotid occlusion or hemorrhage. *Am J Physiol* 1988; **255**: H514–H524.
16. Dobbins DE, Buehn MJ, Dabney JM. Constriction of perfused lymphatics by acetylcholine, bradykinin and histamine. *Microcirc Endoth Lymphatics* 1990; **6**: 409–425.
17. Dabney JM, Buehn MJ, Dobbins DE. Perfused prenodal lymphatics are constricted by prostaglandins. *Am J Physiol* 1991; **260**: H1–H5.
18. Dobbins DE, Dabney JM. Endothelin-mediated constriction of prenodal lymphatic vessels in the canine forelimb. *Reg Peptides* 1991; **35(1)**: 81–91.
19. Devillier P, Weil B, Renoux M, Menkes C, Pradelles P. Elevated levels of tachykinin-like immunoreactivity in joint fluids from patients with rheumatic inflammatory disease. *N Engl J Med* 1986; **314**: 1323.
20. McDonald DM. Neurogenic inflammation in the respiratory tract: Actions of sensory nerve mediators on blood vessel epithelium of the airway mucosa. *Am Rev Respir Dis* 1987; **136**: S65–S72.
21. Payan DG, Goetzl EJ. Substance P receptor-dependent responses of leukocytes in pulmonary inflammation. *Am Rev Respir Dis* 1987; **136**: S39–S43.
21. Said SI. Influence of neuropeptides on airway smooth muscle. *Am Rev Respir Dis* 1987; **136**: S52–S53.
23. Fuller RW, Conradson T-B, Dixon CMS, Crossman DC, Barnes PJ. Sensory neuropeptide effects in human skin. *Br J Pharmacol* 1987; **92**: 781–788.
24. Dobbins DE, Premen AJ, Soika CY, Dabney JM. Vascular effects of substance P in the canine forelimb. *Microcirculation* 1982; **2**: 161–182.
25. Dobbins DE, Buehn MJ, Dabney JM. Evidence for a $\beta_2$-receptor mediated dilation of canine prenodal lymphatic vessels. *FASEB J* 1990; **4(4)**: A590.

26. Thompson DC, Diamond L, Altiere RJ. Enzymatic modulation of vasoactive intestinal peptide and nonadrenergic noncholinergic inhibitory responses in guinea pig tracheae. *Am Rev Respir Dis* 1990; **142(5)**: 1119–1123.
27. Uchida U, Nomura A, Ohtsuka M *et al.* Neurokinin A as a potent bronchoconstrictor. *Am Rev Respir Dis* 1987; **136**: 718–721.
28. Lundin L, Norheim I, Landilius J, Oberg K, Theodorsson-Norheim E. Carcinoid heart disease: Relationship of circulating vasoactive substances to ultrasound detectable cardiac abnormalities. *Circulation* 1988; **77**: 264–269.

ACKNOWLEDGEMENT. This work was supported by USUHS grant #RO 76 DD.

**Received 17 April 1992; accepted in revised form 10 June 1992**
